# Model-predicted geometry variations to compensate material variability in the design of classical guitars

**DOI:** 10.1038/s41598-023-37943-y

**Published:** 2023-08-07

**Authors:** Alexander Brauchler, Sebastian Gonzalez, Manuel Vierneisel, Pascal Ziegler, Fabio Antonacci, Augusto Sarti, Peter Eberhard

**Affiliations:** 1 Institute of Engineering and Computational Mechanics, University of Stuttgart, Stuttgart, Germany; 2 Musical Acoustics Lab at the Violin Museum of Cremona, DEIB-Politecnico di Milano, Cremona Campus, Cremona, Italy

**Keywords:** Engineering, Mechanical engineering

## Abstract

Musical instrument making is often considered a mysterious form of art, its secrets still escaping scientific quantification. There is not yet a formula to make a good instrument, so historical examples are regarded as the pinnacle of the craft. This is the case of Stradivari’s violins or Torres guitars that serve as both models and examples to follow. Geometric copies of these instruments are still the preferred way of building new ones, yet reliably making acoustic copies of them remains elusive. One reason for this is that the variability of the wood used for instruments makes for a significant source of uncertainty—no two pieces of wood are the same. In this article, using state-of-the-art methodologies, we show a method for matching the vibrational response of two guitar top plates made with slightly different materials. To validate our method, we build two guitar soundboards: one serving as a reference and the second acting as a copy to which we apply model-predicted geometry variations. The results are twofold. Firstly, we can experimentally validate the predictive capabilities of our numerical model regarding geometry changes. Secondly, we can significantly reduce the deviation between the two plates by these precisely predicted geometry variations. Although applied to guitars here, the methodology can be extended to other instruments, e.g. violins, in a similar fashion. The implications of such a methodology for the craft could be far-reaching by turning instrument-making more into a science than artistic craftsmanship and paving the way to accurately copy historical instruments of a high value.

## Introduction

One of the largest problems found in guitar making is the lack of reproducibility: even though people talk of a ‘Torres’ or a ‘Hausser’ model^1^, intrinsic material variations of the wood make every instrument unique. Yet, in their uniqueness, they all share a certain *something* that lets us speak of the different models and their characteristic sounds. To achieve that characteristic sound, luthiers subtly modify the internal bracing of the guitar based on their experience and sensibility. This is where the art comes in.

There are, however, many reasons why a more systematic approach to instrument-building is timely and sorely needed. Global warming has already altered the habitat of trees^2,3^, and tone-wood (the particular kind of spruce used for the soundboard of musical instruments) is bound to become more and more scarcely available. Recent research has clearly shown that the design of a guitar is much more important for the sound than environmental and material variability^4^. What this research lacks, however, is a concrete methodology of how to compensate for those material variations by adjusting the design. In this article, we close this gap by combining the state-of-the-art in parameter identification applied to guitars together with standard optimisation techniques.

The starting point for our research lies with the recent advances in simplified FEM simulations for musical instruments, be they for guitars with model order reduction methods^5,6^ or violins with neural networks^7^. These simplified approaches allow us to obtain the values for the eigenfrequencies of the system in 1/1000th of the time compared to traditional FEM simulations without significant loss of accuracy, allowing us to perform optimisations in a reasonable time^6,8^. The traditional approach has been used in a variety of musical instruments, from the kantele^9^ to the viola da gamba^10^, with numerous studies focusing on string instruments^4–8,11–20^.

In engineering, shape optimisation using finite element models evolved into a standard method used in many applications^21,22^. However, in the field of musical instruments, the only examples that we are aware of are the shape optimisation of a bell, e.g.^23–25^, of vibraphone bars^26–28^, and a simplified model of the violin top plate^29^. To the best of the authors’ knowledge, shape optimisation has not yet been applied to string instruments because, in stark contrast to other examples, one does not know how to choose a suitable objective function because one lacks objective criteria to define a ‘good’ instrument. We can, however, count on the expert knowledge of luthiers and the evaluation of musicians to identify ‘good’ examples of guitars or violins^30–32^. Therefore, trying to copy the vibrational response of these well-sounding instruments seems to be the most reasonable choice when it comes to designing an objective function for the optimisation of string instruments like guitars.

**Figure 1:**
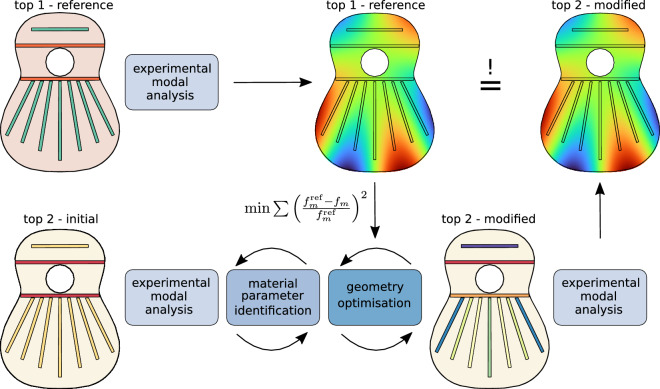
Visualisation of the workflow that we use for matching the modal parameters of two soundboards by compensating the material differences through geometry modifications. Starting from the modal parameters of the reference plate (top 1). By fitting the material parameters of a different plate (top 2) in an initial state with thicker braces than the reference, we can optimise the geometry of the bracing by only looking at the differences in the eigenfrequencies. Finally, the experimental modal analyses show that two different geometries can have a very similar vibrational response.

This article’s contribution is a methodology for creating a vibrational copy of a reference guitar’s top plate. We focus on the top plate since it is not only the most complex but also the most relevant part for sound production in a guitar. Figure 1 shows a diagram of the proposed methodology: Starting from a reference plate (top 1), we identify its modal parameters—namely eigenfrequencies, eigenmodes, and modal damping ratios—in a frequency range up to 1000 Hz. This frequency range has been chosen since up to this frequency our experimental results are admissible. Beyond that, the mesh we used to compute the modal parameters is too coarse to correctly identify the modes. The measured modal parameters of another plate (top 2) with identical geometry besides initially higher braces are fed into a material parameter identification process to obtain a virtual prototype that is able to predict the influence of geometric changes on this plate. This virtual prototype is used in numerical optimisation of the bracing heights to compensate for the differences in the eigenfrequencies between the two plates. The modifications to the bracing height are then applied to top 2 as predicted by the numerical optimisation. Finally, the experimental modal analysis of the reference and the modified copy shows that two different geometries can have a very similar vibrational response.

We believe this is an excellent starting point to tackle more general problems regarding the interaction between material and design of musical instruments, as it demonstrates that the traditional geometrical reconstruction of the outer shape of instruments is not necessarily the best to obtain a standard vibrational response. Our approach shows that noticeable geometric modifications must be considered if a reference plate’s vibrational response is to be achieved. Even though the timbre of the instrument is also dependent on the high frequency range^33^, a modal approach is not useful here as the modal overlap becomes relevant and a statistical approach should be used instead^34^.

## Results

We constructed two guitar top plates according to a simplified Torres fan bracing pattern^1^. Great care was taken in building them as similarly as possible by matching the density of the braces used and the final weight of the top plates.

A diagram of the two plates and their bracing height indicated by colours can be seen in Fig. 1, for ‘top 1-reference’ and ‘top 2-initial’. We have coloured the top plates in different shades to indicate that they have different material parameters. In particular for top 1 the identified longitudinal stiffness is $E_{1L} = 11.6$ GPa and the density is $\rho _1=403\,\mathrm {kg\,m^{-3}}$ and for top 2 we get $E_{2L}=9.31$ GPa and $\rho _2=407\,\mathrm {kg\,m^{-3}}$. In total, 35 material parameters are identified for each top as all braces are handled individually (see “Methods”). We have started from very thick braces (7 mm in the fan region) for two reasons. First, it serves us as a validation of the numerical model for different geometric configurations. Secondly, when we try to optimise top 2 to fit the vibrational response of top 1, we need to have a range of possible heights for the braces, and since taking out wood is easier than adding it, we decided to start from an oversized configuration.

**Figure 2:**
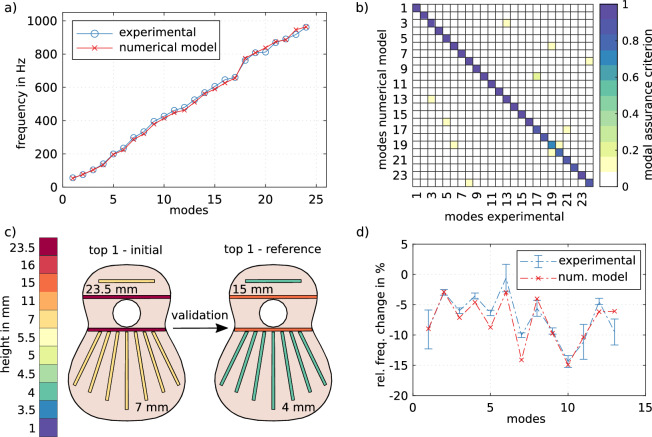
(**a**) Comparison of eigenfrequencies calculated from the numerical model and experimentally identified eigenfrequencies for the initial configuration of top 1. (**b**) Comparison of eigenmodes between the numerical model and the experimental modal analysis. (**c**) Conducted bracing height changes to validate the numerical model. (**d**) Relative frequency changes caused by the bracing height variation in the numerical model and the experiment.

Figure 2a shows the eigenfrequencies of the experimental setup and the numerical model once the material parameters of the braces and the plate have been identified for top 1. The experimentally identified eigenfrequencies are the mean values from 4 measurements, with the eigenfrequencies varying in a mean range of $\pm 0.7\%$ for the first 24 modes. Figure 2b shows the modal assurance criterion matrix^35^ for the first 24 modes of the top plate—showing that the modal similarity is excellent. In order to validate the model, we implement the modal identification in two stages. Starting from the initial configuration with rather thick braces (Fig. 2c top 1-initial), we arrive at the reference configuration (Fig. 2c top 1-reference) and compute the relative change of the eigenfrequencies in the numerical model as well as in the experimental setup. Figure 2d shows the relative change in the frequencies for the experiments and simulations. Notice that the material parameter identification is only made for the initial configuration of top 1. Further insight into the error of the numerical model is given in the Supplementary Material.

**Figure 3:**
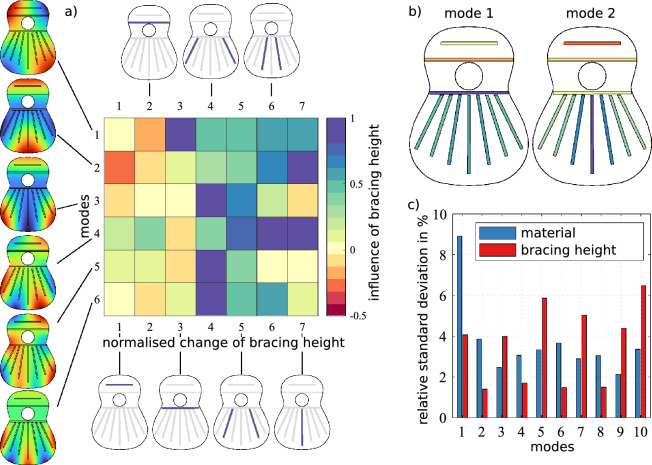
(**a**) Correlation matrix between eigenfrequencies of the first modes and the height of specific bracing areas. (**b**) Bracing height changes that are necessary to change the eigenfrequency of the specified modes (colors match the colorbar in Fig. 3a). (**c**) Relative standard deviation of eigenfrequencies caused by feasible bracing height changes and possible material parameter changes within spruce wood.

Once the material parameters are fully identified, we can develop a linear regression model of the relative influence of the bracing height on the eigenfrequencies (see “Methods”). Figure 3a shows the correlation between the braces’ heights and the eigenfrequencies of the first 6 modes of the top plate. In fact, we find the dependency of the eigenfrequencies on the bracing heights to be approximately linear for the first 13 modes (see Supplementary Material). The vertical inset shows the modal shape associated with each eigenfrequency, whereas the horizontal diagrams show which of the braces it refers to. Notice that for symmetry, we only take pairs of braces in the fan, except for the central one. In Fig. 3b, the height changes necessary to influence the eigenfrequency of modes 1 and 2 are depicted vividly.

To quantify the variability in the eigenfrequencies due to material or design parameter changes, we sample the parameter space for each of them (geometric and material) in the range of possible values for each (see “Methods”). We sample the eigenfrequencies 10,000 times and obtain a distribution that turns out to be very close to normal, similarly to the results reported in Ref.^7^. From those distributions, and for each eigenfrequency, we measure the standard deviation of the frequency change. Figure 3c shows the relative standard deviation in percentage for the first 10 modes of the top plate. Interestingly, the variation due to material parameters is, on average, slightly larger than that due to geometric variations, which explains why the wood selection is such a critical step in instrument making. Particularly modes 1, 2, 4, 6, and 8 are extremely sensitive to the variation in the material parameters and are much less affected by the bracing height. We conclude that these modes depend more on the plate’s specific stiffness than on the braces’ geometry. Nevertheless, these results explain that our method to reproduce the vibrational response works well since we started with very similar woods that only vary in their stiffness and had almost the same density.

Finally, in order to find out the necessary bracing heights of top 2 so that it vibrates as top 1, we use a straightforward optimisation process. Instead of using directly the finite element simulations at each iteration step, we use the linear regression model (explained in detail in the “Methods” section and the Supplementary Material) to solve the optimisation problem of the eigenfrequencies of both top plates with the objective function

1 $$\begin{aligned} {\fancyscript{L}} = \sum _{m=1}^{13} \left( \frac{f_m^{\text {ref}}-f_m}{f_m^{\text {ref}}}\right) ^2. \end{aligned}$$

Note that this function must be normalised so as to only compare relative changes in frequency and not give excessive importance to the higher modes. The number of modes used to define this function is arbitrary, but the higher the number of modes included, the more their shapes change, and the purely frequency-based objective function becomes less effective. We have settled on 13 as the largest number of modes that still behaves well. To minimise this objective function, we use Matlab’s *fmincon* constrained optimisation algorithm for the seven brace heights shown in Fig. 3a.

**Figure 4:**
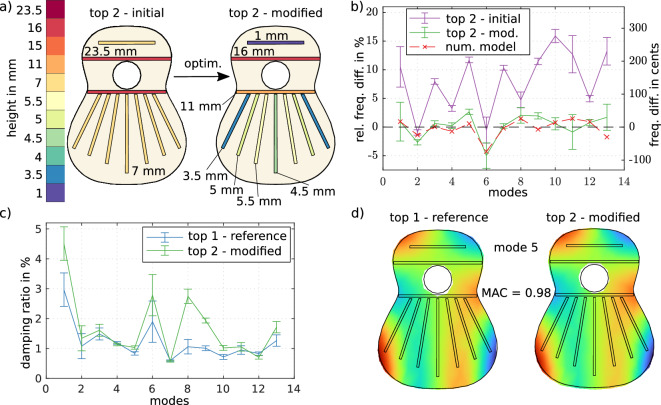
(**a**) Conducted bracing height changes given by the optimisation to fit the soundboard copy to the reference. (**b**) Experimentally identified relative frequency difference compared to the reference soundboard before and after the height changes. (**c**) Modal damping ratios of the reference and the copy soundboard in comparison. (**d**) One exemplary experimentally identified mode in comparison between reference and copy.

The optimisation problem is considered to be converged if the first order optimality criterion $||\nabla {\fancyscript{L}}|| < 10^{-10}$ is reached. Around 100 iterations are necessary until convergence. Although several starting points have been tested, it cannot be ruled out completely that the presented solution is a local, rather than a global, minimum. The resulting height profile for the braces is carved into top 2 as depicted in Fig. 4a. After the optimisation, the difference in eigenfrequencies between top 1 and top 2 can be observed for the initial and modified configuration (purple and green line in Fig. 4b respectively), as well as the difference predicted from the numerical model (red line). The mean error between the eigenfrequencies of top 1 and top 2 after modification is 1.55% or 27 cents (roughly the quarter of a semitone), which is extremely well predicted by the numerical model. The maximum error between eigenfrequencies of the tops is 4.99% or 88 cents for the sixth mode, which is still below one semitone difference.

**Figure 5:**
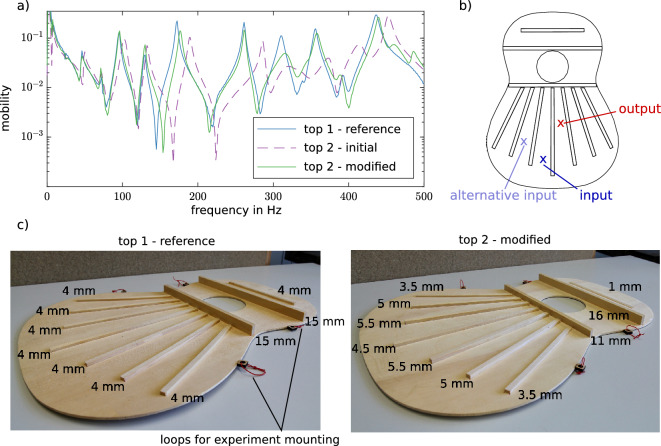
(**a**) Measured frequency response functions for the plates in comparison. (**b**) Input and Output position of the mobility in (**a**) and alternative input that is used for the modal analysis. (**c**) Pictures of the two plates in their final configuration with depicted bracing heights next to the braces. Especially for the harmonic bars, the height difference between the corresponding braces of the two plates is clearly visible.

While the modal shapes are primarily influenced by the shape of the tops, the modal damping ratios are mainly a material property and are expected not to change much if the bracing heights are changed. Hence, our objective function only considers the eigenfrequencies, for they are the main quantities that have to be adjusted. Figure 4c shows the damping ratio for the reference top 1 and the optimised top 2. The match is very good for the first six modes, so we expect a similar tonal performance of both top plates when used in a complete instrument. However, the damping ratios of the eighth and the ninth eigenmode deviate considerably. This difference is also visible before modification of top 2 and, hence, is not caused during the optimisation process. From the acquired data, the cause of this deviation cannot be explained unambiguously. Possible causes might be uncertainties in the manufacturing process, e.g. the glued connections, or the position of the experimental mounting. A locally inhomogeneous material might be a further possible cause. The fit of the modal shapes is even better, showing an average MAC of 0.92 for the first 13 modes. An example of the mode similarity can be seen in Fig. 4d where for mode 5, a MAC of 0.98 is achieved. In conclusion, by fitting the first 13 eigenfrequencies, our model can correctly predict a height profile that top 2 needs to yield a similar vibrational response to top 1. The results are also excellent for both modal shapes and damping ratios, which come ‘for free’ when optimising the eigenfrequencies. From modal analysis theory, it is expected that matching modal parameters should result in a similar vibrational response^36^. The graphs depicted in Fig. 5a confirm this expectation for one exemplary measured mobility compared between top 1 in its reference state and top 2 before and after the modification. The modified top 2 has a very similar frequency response to the one of top 1. In Fig. 5c the experimental plates are shown in their final state. The height differences between the harmonic bars are clearly visible while the small height differences in the fan are barely recognisable.

## Discussions

In this article, we have presented a methodology for the material identification of a guitar top plate and a predictive framework that allows one to ‘copy’ a target vibrational response. The framework uses state-of-the-art methodologies to rapidly compute a linear regression model of the vibrational response of the plate as a function of the bracing height (see Supplementary Material). We then use that linear regression model to minimise an objective function based on the first 13 eigenfrequencies to obtain the geometry that matches the vibrational response in a top plate of a different material than the original. We carve the optimised bracing height profile in our experimental top and show that, indeed, the results are very similar, not only for the eigenfrequencies but also for the damping ratios and the modal shapes of the first 13 modes. Note, however, that the damping ratios cannot yet be explicitly controlled with the methodology since they are not included in the objective function. The decay time of the sound, for example, is one of the most important perceptual characteristics of an instrument, and our method is not able to optimise for this in its current formulation. Compared to previous results, however^8,29^, our method has been shown to work experimentally, demonstrating, for the first time, that such computational approaches can be used in real life for the deliberate construction of vibrational copies of musical instruments. The question of whether fitting only the low-frequency modes ($\le \; 500$ Hz) is enough to reproduce the timbre of the instrument or if the error achieved is perceptually significant is still open and requires further study.

Thanks to the model order reduction technique, we can sample a vast array of material and geometrical values and study how geometry and material parameters affect the eigenfrequencies. Previous results on the use of metamaterials for thin wooden plates^37^ show that, to a certain degree, the density and stiffness of the plates can be purposefully controlled. By matching the density and the longitudinal stiffness of the top and the back plate, we can be certain that the modifications of the braces will be minimal^6^.

The implications of these results are far-reaching in the field of instrument making: By quantifying how close we can come to a desired vibrational response with a given material, we have taken guesswork out of the equation in guitar-making. One could argue that the equipment used in this research is far beyond the reach of standard contemporary instrument-making workshops. However, the principles behind our method can be applied with any setup of modal identification. Some luthiers already perform this as part of their workflow to characterise their instruments^38–40^. Furthermore, recent advances in the development of efficient surrogate models like neural network prediction of the vibrational response of wood^7,41^ and parametric model order reduction for shape optimisation^42^ make us hopeful that in the near future this optimisation method can become a fundamental part of guitar-makers’ toolboxes.

Finally, the ability to deliberately achieve a certain vibrational response is unheard of in classical instrument making. Instead of blindly following older designs or searching for new ones based only on intuition, we propose a method for scientifically and methodically producing a copy of a given instrument. This is not only relevant in guitar-making but in other instruments as well, and opens new possibilities for the construction of facsimiles and acoustic copies of historically relevant instruments^43–45^. A case in point is violins, where some historical instruments more than 300 years old are no longer suited for actual playing. This method could help us hear these instruments (and the example of Stradivari’s Messiah comes immediately to mind) by creating accurate acoustic copies of them. Whether the accuracy of the method is sufficient to produce perceptually^32,46,47^ indistinguishable copies of instruments still remains an open question. The path is rather long still, but this is a necessary step in the direction of turning instrument making more into a science than a mysterious art.

## Methods

### Guitar plates construction

The guitar plates were built with same-grade wood pieces bought from the same dealer (Rivolta Wood, Desio, Italy). The wood used is Abete Rosso (*Picea abies*). Each guitar plate is made of two bookmatched pieces supplied by the dealer. The plates were glued up with fish glue (Kremer pigments, Aichstetten, Germany) using the traditional guitar-making methods. Six weeks passed between glueing the top plate and glueing the braces to allow the wood to regain its original moisture content.

The wood for the braces was sorted by density, and the two top plates were matched as closely as possible to have the same weight distribution on space. The mass variation of the braces for either guitar was less than 3 gr. Due to the size of the brace wood, an independent material parameter identification could not be used, and we opted for a bulk characterisation from the reduced-order model. The braces were glued to the soundboard with rabbit glue (Cremona tools, Cremona, Italy). The braces were planed by hand to a standard cross-section of 7 $\times$ 7 mm and glued in a fan pattern based on a simplified Torres model from 1884^1^, the Stradivari of guitar-making. Harmonic bars had a 23.5 $\times$ 7 mm cross-section. Before glueing the fan bars, we measured their density and ordered them in such a way that the heaviest bars were in the centre of the top plate and the lightest on the sides. This was done to have the two top plates as similar as possible in terms of their weight distribution. The impact of the glue was in no way characterised and assumed irrelevant. After construction, the plates were shipped from Cremona to Stuttgart, where they were kept in a climate-controlled room for 3 months before starting the measurements.

### Experimental modal analysis

Experimental modal analysis is the standard method when it comes to identifying modal parameters of vibration structures and has been applied to various musical instruments^5,36,40,48,49^. A setup with the guitar plates being suspended by very soft springs approximating free boundary conditions was developed for the experimental modal analysis. The plates were kept, and measurements were taken in a climate-controlled room, with a relative humidity of $55\pm 1\%$ and a constant temperature of $24\; ^{\circ }$C. One can find details on the climate-controlled room in Ref.^50^. The plates’ velocities were measured with a Polytec PSV-500 Scanning Laser Doppler Vibrometer, and an automatic impulse hammer acts as the excitation device^51^. The excitation with the hammer yielded a reproducible excitation of frequencies up to 1000 Hz, and the maximum forces on the guitar plates did not exceed 3.0 N. A total of 220 mobilities were measured. These measurements were composed of 110 points on the soundboard, where the velocity is measured, and two distinct excitation positions between the fan braces as depicted in Fig. 5b. Each measurement resulted in a data sequence of duration $T = 0.8$ s. Using a sampling step of $\Delta t=1.6 \times 10^{-4}$ s, the width of each frequency bin of the corresponding Fourier transform was $\Delta f=1.25$ Hz. Longer measurements would have resulted in zero padding due to the faded signal and were, therefore, avoided. The complex mode indicator function, in combination with enhanced frequency response functions, was used to identify the modal parameters of the plates. Details on the method can be found in^52^, and a detailed description of the application to a classical guitar is included in^5^. The uncertainty of the modal parameter changes, given in the error bars in Figs. 2 and 4, was calculated from 15 measurements throughout the modification process of top 1 by interval arithmetic^53^.

### Material parameter identification

The material parameter identification follows the approach described in detail in Ref.^6^. Detailed finite element models of the guitar plates act as the key pieces of the approach^54,55^. The models were created in the commercial software Abaqus with free boundary conditions and an orthotropic material model for all the braces^56^. The plates were discretised with linear shell elements of Abaqus type S4, while the braces’ discretisation was carried out with linear C3D8 volume elements. In former publications, rigid tie constraints have shown to be a reasonable assumption for binding the plate and the braces together^5,6^. Hence, this approach was used in this publication, too. The degrees of freedom of the full-order model with a very fine discretisation sum up to $N=400128$.

Unfortunately, the detailed model takes too much computational time to be evaluated thousands of times during the parameter identification procedure. Furthermore, the parameter space would contain up to 107 material parameters if all braces with all their material parameters were to be identified individually. Thus, a projection-based Krylov approach for parametric model order reduction was applied to reduce the number of degrees of freedom in an efficient surrogate model to $n=600$ while keeping a good approximation of the full-order model’s results up to 1000 Hz. This was reached by matching the transfer function of 4 inputs and outputs distributed over the plate at 20 frequency shifts equally distributed in the frequency range and 60 parameter expansion points created with a Sobol sequence as explained in^6^. General information on model order reduction can be found in Refs.^57,58^ while a review of parametric model order reduction techniques is given in Ref.^59^, and the used software is described in Ref.^60^. The order-reduction approach reduces the computational time to calculate the first 30 modal parameters from 78 s with the full-order model to 0.04 s with the reduced-order model, corresponding to a numerical speedup of 1950. On a set of test data consisting of 100 evaluations for the first 30 eigenfrequencies, the common coefficient of determination is $R^2=0.96$ between the reduced-order model and the full-order model^61^.

In the reduced-order model, parameter dependency is preserved for the 35 most influential parameters chosen with the help of a sensitivity analysis (see Supplementary Material). The parameters kept for the plates are the density $\rho$, the Young’s moduli $E_{\textrm{L}}$ in longitudinal and $E_{\textrm{R}}$ in the radial direction, and the shear modulus $G_{\textrm{LR}}$. The parameters are identified for each brace individually. However, the number of parameters varies between the different braces as follows. Parameters for the fan braces comprise $\rho$, $E_{\textrm{L}}$, and the shear modulus $G_{\textrm{LT}}$ with the subscript *T* denoting the tangential direction. The higher braces directly above and below the soundhole are characterised by $\rho$, $E_{\textrm{L}}$, the Young’s modulus in tangential direction $E_{\textrm{T}}$, and $G_{\textrm{LT}}$. The horizontal brace on the upper part of the plates is parameterised with $\rho$ and $E_{\textrm{L}}$. Values for spruce from Ref.^62^ are used for all the remaining values.

The parameter identification procedure follows a two-step approach. Firstly, the 35-dimensional parameter space were explored as done in Ref.^6^ using a sampling approach based on a Sobol-sequence and one million samples^63^. In the second step, an objective function comparing eigenfrequencies and eigenmodes was evaluated, and the eight best-performing solutions were given into the Matlab *fmincon* algorithm as starting values. In both sets, constraints were set in such a way that the total mass of the plates would not allow variations beyond $\pm 5\%$ with respect to the experimentally measured value. As a second set of constraints, the material parameter values were not allowed to exceed bounds taken from literature^4,62,64,65^. The best solution with respect to the objective function evaluated for the first 24 modes of the algorithm was used for the geometry optimisation.

### Geometry optimisation

The finite element model with the identified material parameters served as a virtual prototype to apply the changes to the braces. The model of top 1 was used for validation purposes, as shown in Fig. 2, while the model with the material parameters for top 2 was used to optimise the bracing heights of top 2 to match the modal parameters of top 1, as visible in Fig. 4. In this procedure, seven independent height parameters were used for the ten braces as the symmetry in the fan braces was kept constant. Again, the full-order model turned out to be unsuitable for optimisation purposes due to its high computational cost. For this reason, a linear regression model was fitted for the correlation between the first 13 eigenfrequencies and the heights of the braces. The linear regression model was trained with a set of 950 parameter samples of bracing heights created from a Sobol sequence in a realistic range of $h_{\textrm{range}}=\left[ 1\,\textrm{mm},7\,\textrm{mm}\right]$ for the lower braces and $H_{\textrm{range}}=\left[ 8\,\textrm{mm},23.5\,\textrm{mm}\right]$ for the harmonic bars. The resulting linear regression model is given in the Supplementary Material. On a set of test data consisting of 50 further samples, the coefficient of determination is $R^2=0.96$.

This regression model was then used in the optimisation process to identify the optimal bracing heights. Since the finite element model approximates the absolute values of the eigenfrequencies with a small systematic error, the desired relative change of eigenfrequencies was used as optimisation criterion as depicted in the violet curve in Fig. 4b. Hence, the systematic error between the finite element model and the experiment did not influence the results. Then, the mean squared error of the first 13 eigenfrequencies was minimised using Matlab’s *fmincon*. The only constraints set in the optimisation process were the lower and upper bounds, and they were specified as written above in $h_{\textrm{range}}$ and $H_{\textrm{range}}$.

### Supplementary Information

Supplementary Information.

## Data Availability

The datasets generated during and/or analysed during the current study are available from the corresponding author upon reasonable request.
