# Supplementary material for: Model-predicted geometry variations to compensate material variability in the design of classical guitars
Source: Sci Rep. 2023 Aug 7;13:12766. doi: 10.1038/s41598-023-37943-y (PMC10406898; doi:10.1038/s41598-023-37943-y)
Supplement: Supplementary file 1 — Supplementary Information. [file 41598_2023_37943_MOESM1_ESM.pdf]

Supplementary Material for 'Model-predicted geometry variations to compensate material variability in the design of classical guitars'

Alexander Brauchler[1,*], Sebastian Gonzalez[2,+], Manuel Vierneisel[1], Pascal Ziegler[1], Fabio Antonacci[2], Augusto Sarti[2], and Peter Eberhard[1]

[1]Institute of Engineering and Computational Mechanics, University of Stuttgart, Stuttgart, Germany
[2]Musical Acoustics Lab at the Violin Museum of Cremona, DEIB - Politecnico di Milano, Cremona Campus, Italy
[*]alexander.brauchler@itm.uni-stuttgart.de
[+]tsuresuregusa@gmail.com

ABSTRACT

This document contains details on the sensitivity analysis conducted before the material parameter identification of the two guitar tops and further information on the linear regression model used for the optimisation of the bracing heights. Furthermore, a more detailed comparison of the damping between the two plates is given, and the differences between the numerical model and the experimentally identified values are discussed.

Sensitivity analysis

A sensitivity analysis was carried out before the parameter identification to only include the most influential material parameters in the material parameter identification. During this analysis, the braces were assumed to have the same material parameters. In the final identification procedure, however, they were treated individually, as described in the main text of the article. Hence, the material parameters comprise the density ρ as well as the Young's moduli (E_{L}, E_{R}, E_{T}), the Poisson ratios (ν_{LR},ν_{LT},ν_{RT}), and the shear moduli (G_{LR},G_{LT},G_{RT}) in all three directions for the plate (superscript pl) and for all the braces combined (supersicrpt br). The sensitivity analysis is conducted by creating 800 samples with a Sobol set for the material parameters. Then, we calculate the correlation coefficient between the material parameters and the first 13 eigenfrequencies. The correlation coefficient $\Gamma(x,y)$ is a measure for the linear dependence of two random variables x and y in a number of $\tilde{N}$ random experiments. It is defined as

$$\Gamma(x,y) = \frac{1}{\tilde{N}-1}\sum_{\tilde{n}=1}^{\tilde{N}} \frac{(x_{\tilde{n}}-\zeta_{\mathrm{x}})(y_{\tilde{n}}-\zeta_{\mathrm{y}})}{\psi_{\mathrm{x}}\psi_{\mathrm{y}}} \tag{1}$$

where ζ is the mean and ψ is the standard deviation. The most important parameters and their influence are depicted in SFig. 1.

Linear regression model

In SFig. 2, the matrix $\boldsymbol{A} \in \mathbb{R}^{13\times 11}$ of the linear regression model is displayed that was fitted to approximate

$$\begin{bmatrix} f_1 \\ f_2 \\ \vdots \\ f_{13} \end{bmatrix} = \underbrace{\begin{bmatrix} a_{11} & a_{12} & \cdots \\ a_{21} & a_{22} & \cdots \\ \vdots & \vdots & \ddots \\ & & \end{bmatrix}}_{\boldsymbol{A}} \begin{bmatrix} 1 \\ h_{\mathrm{a}} \\ h_{\mathrm{b}} \\ \vdots \\ h_{\mathrm{j}} \end{bmatrix} \tag{2}$$

with $h_{\mathrm{a}},\cdots,h_{\mathrm{j}}$ denoting the heights of the different braces and the eigenfrequencies $f_1,\cdots,f_{13}$.

The relative error

$$\varepsilon = \frac{f_m^{\mathrm{reg}} - f_m^{\mathrm{FE}}}{f_m^{\mathrm{FE}}} \tag{3}$$

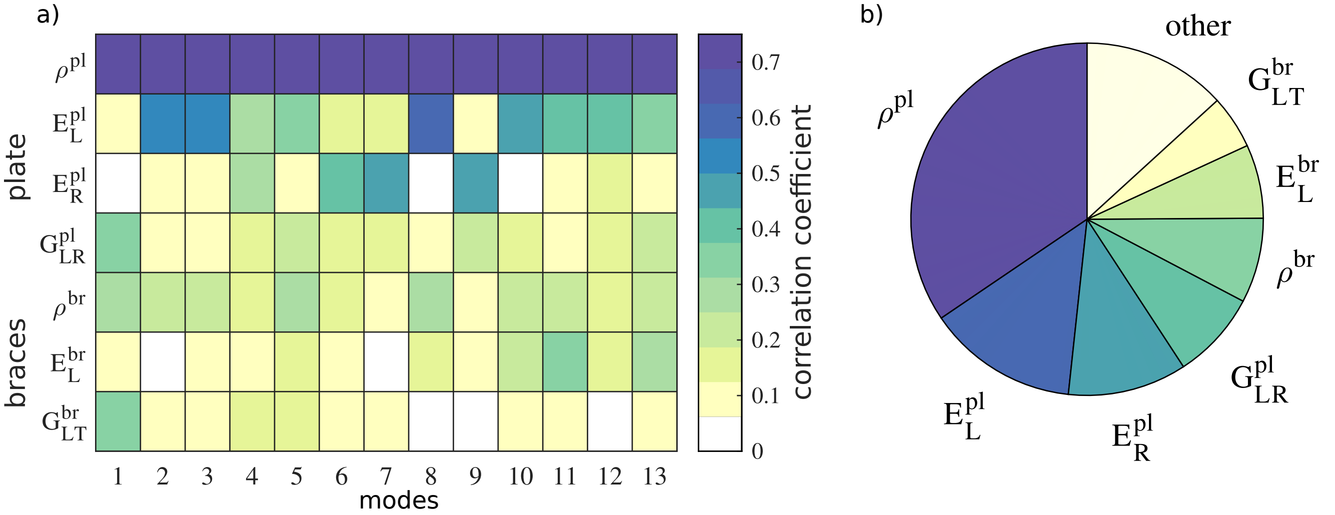

Supplementary Figure 1. (a) The correlation coefficient between the most influential material parameters and the first 13 eigenfrequencies. (b) 2-norm of the correlation coefficient over the first 13 eigenmodes arranged as a pie plot to compare the influence of the material parameters.

of the eigenfrequencies calculated with the regression model f_m^{reg} and the eigenfrequencies of the finite element model f_m^{FE} are visualised with box plots in SFig. 3. The boxplot represents the error data with median, the 0.25 quantile, the 0.75 quantile, outliers, as well as the minimum and the maximum values that are not outliers. The lower and upper end of the box represent the 0.25 and the 0.75 quantiles, respectively, and the horizontal line in the box depicts the median of the data set. The lines that extend below and above each box, illustrate the nonoutlier minimum and the nonoutlier maximum in the data, respectively. Points are defined outliers if they lie more than 1.5 times the height of the box above or below the box and are depicted as dots in the diagrams. Almost all samples for the first 13 modes come with $\varepsilon < 1\,\%$. This justifies the choice of the linear regression model during the optimisation process. Note, however, that a better regression model should be used if more modes are taken into consideration as the error increases for the 14th and 15th mode.

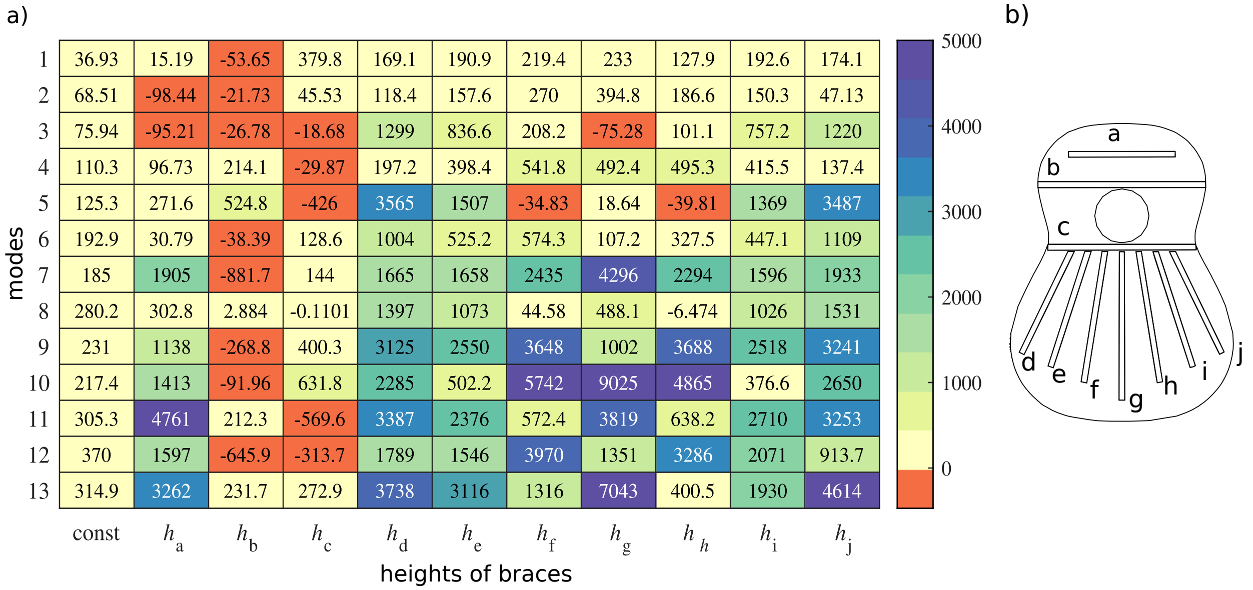

Supplementary Figure 2. (a) Matrix $\boldsymbol{A}$ defining the linear regression model. (b) Soundboard with the letters referring to the heights of the braces in (a).

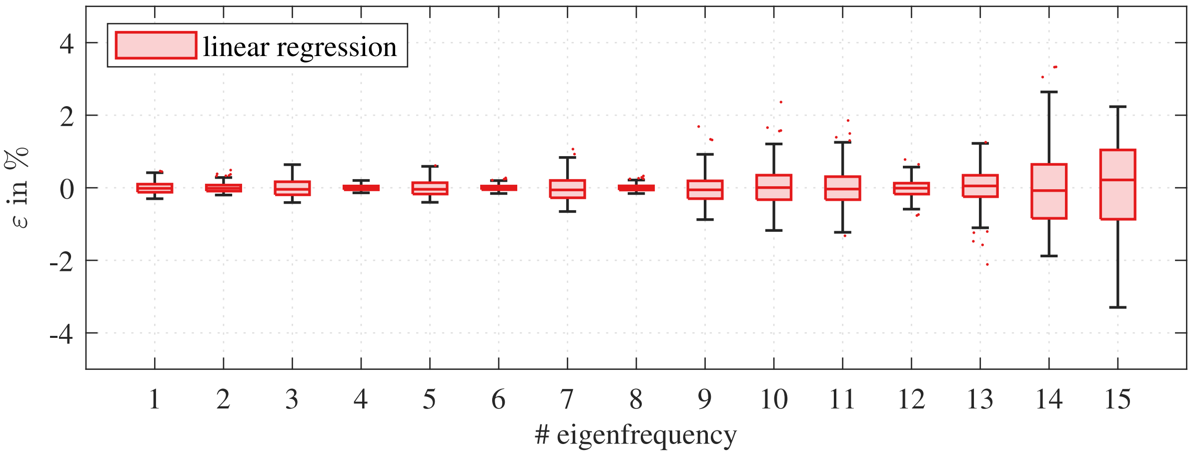

Supplementary Figure 3. Boxplots for the relative error introduced with the linear regression model for 200 samples of different parameter heights that were not used during training of the model.

Damping and decay times

In Fig. 4c of the main manuscript, the modal damping ratios of the two tops in their final state are displayed. For a better idea on how these modal damping ratios influence the time domain, we present two further figures here. First and foremost, SFig. 4 depicts the measured time signals of the two plates corresponding to the frequency response presented in Fig. 5a in the main manuscript. The very similar time decay of the two signals shows the similarity in the damping properties of the two plates. Furthermore, from the identified modal damping ratios, the exponential time decay per mode can be computed as

$$x_m(t) = x_0 \exp\left(-2\pi f_m \zeta_m t\right) \quad (4)$$

with the modal damping ratios ζ_m, the eigenfrequencies f_m, the time t, and the initial amplitude x_0. The whole transient decay can be computed by summing up the modal transients. This exponential time decay is displayed in SFig. 5 for the first eight modes with the identified values for top 1 and top 2 in their final states to give the reader an impression of the time decay of the different modes. This is nothing but the time domain representation of the results presented in Fig. 4c of the main manuscript, yet gives a more tangible idea of the modal similarity of the two tops.

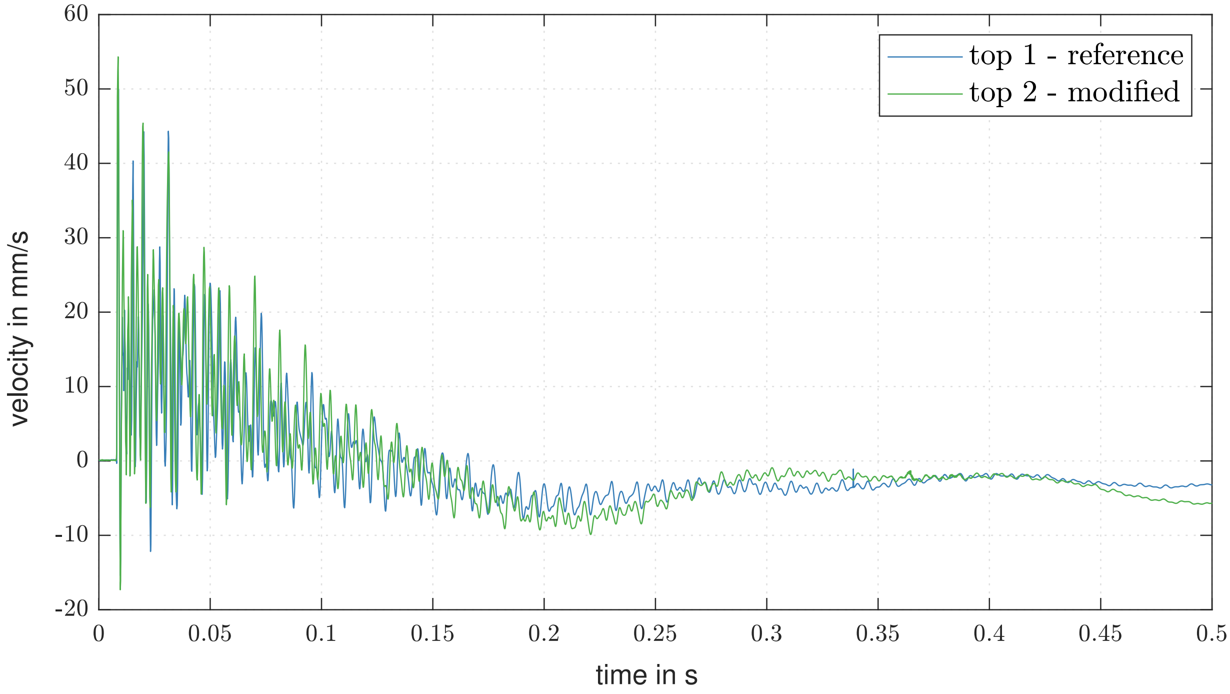

Supplementary Figure 4. Measured velocity over time signal corresponding to the frequency response displayed in Fig. 4 of the main manuscript.

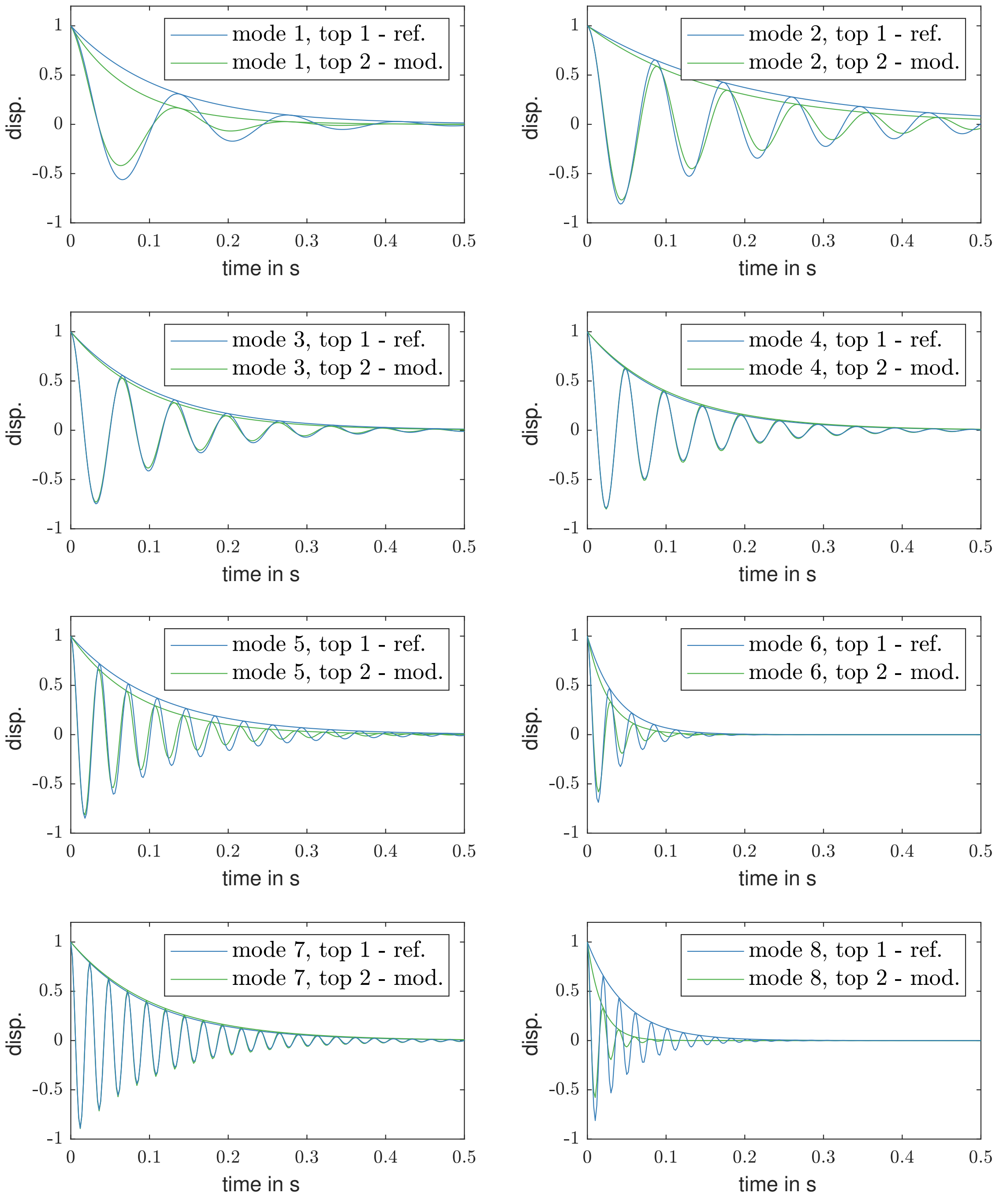

Supplementary Figure 5. Modal decay plots for the first eight modes in comparison between the two plates.

Numerical model error

This section gives additional information on the error of the numerical model with respect to the experimental identification. The data corresponds to the model validation depicted in Fig. 2 of the main article. Supplementary Table 1 contains the first 13 experimentally identified eigenfrequencies and the first 13 eigenfrequencies of the numerical model before (top 1 - init.) and after (top 1 - ref.) the height changes were applied to the braces. Further insight on the model error can be gained from SFig. 6 where the relative error of numerical eigenfrequencies f_m^{num} with respect to the experimental eigenfrequencies f_m^{exp}

$$\varepsilon_{\text{rel}} = \frac{f_m^{\text{num}} - f_m^{\text{exp}}}{f_m^{\text{exp}}} \tag{5}$$

for top 1 - init. and top 1 - ref. is given. The error in cents

$$\varepsilon_{\text{cent}} = 1200 \log_2 \left(\frac{f_m^{\text{num}}}{f_m^{\text{exp}}} \right) \tag{6}$$

which is given in SFig. 6, too, is a musically interpretable measure. The errors before and after the modification of the plate are very similar, indicating a good fit of the model and stressing its predictive capabilities. Moreover, the error in cents lies below 100 cents for all modes, i.e., below one semitone.

Supplementary Table 1. Eigenfrequencies of the numerical model and the experimentally identified eigenfrequencies compared for top 1 in the initial configuration and in the reference configuration.

# mode	top 1 - init. freq. experimental	top 1 - init. freq. num. model	top 1 - ref. freq. experimental	top 1 - ref. freq num. model
1	50.24 Hz	53.09 Hz	45.68 Hz	47.53 Hz
2	75.03 Hz	76.87 Hz	72.81 Hz	74.11 Hz
3	101.24 Hz	100.68 Hz	95.19 Hz	93.89 Hz
4	133.97 Hz	132.29 Hz	129.21 Hz	125.21 Hz
5	183.65 Hz	187.73 Hz	171.98 Hz	170.97 Hz
6	225.62 Hz	220.81 Hz	224.27 Hz	212.73 Hz
7	290.39 Hz	283.37 Hz	261.26 Hz	250.22 Hz
8	328.99 Hz	316.22 Hz	310.59 Hz	305.27 Hz
9	375.17 Hz	368.69 Hz	340.16 Hz	327.79 Hz
10	415.41 Hz	408.81 Hz	355.67 Hz	343.20 Hz
11	441.58 Hz	439.27 Hz	392.40 Hz	386.81 Hz
12	456.47 Hz	457.36 Hz	436.05 Hz	428.43 Hz
13	497.71 Hz	491.62 Hz	450.38 Hz	438.71 Hz

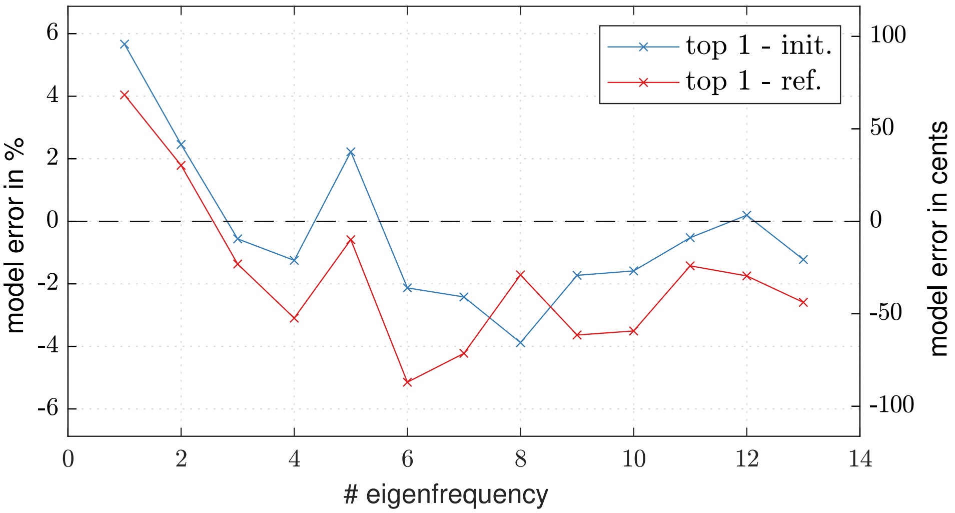

Supplementary Figure 6. Relative frequency error of the numerical model with respect to the experimentally identified eigenfrequencies for the first 13 eigenmodes in the initial state of top 1 and in the reference state of top 1.
